# Population knowledge and awareness of antibiotic use and antimicrobial resistance: results from national household survey 2019 and changes from 2017

**DOI:** 10.1186/s12889-021-12237-y

**Published:** 2021-11-29

**Authors:** Viroj Tangcharoensathien, Sunicha Chanvatik, Hathairat Kosiyaporn, Supapat Kirivan, Wanwisa Kaewkhankhaeng, Apichart Thunyahan, Angkana Lekagul

**Affiliations:** 1 International Health Policy Program, Ministry of Public Health, Nonthaburi, Thailand; 2 National Statistical Office, Ministry of Digital Economy and Society, Bangkok, Thailand

**Keywords:** Knowledge, Awareness, Antibiotic use, Antimicrobial Resistance, General population, Thailand

## Abstract

**Background:**

Lack of knowledge and awareness on antimicrobial resistance (AMR) can result in irrational use of antibiotics, which is one of the major drivers of AMR. One goal of the Thailand National Strategic Plan on AMR (2017-2021) is a 20% increase in public knowledge and awareness of antibiotic use and AMR by 2021. This study assesses antibiotic use, level of knowledge and awareness of antibiotic use and AMR and the factors associated with their knowledge and awareness in the Thai population in 2019. It compares findings with a similar national survey in 2017.

**Methods:**

An AMR module was integrated into the Health and Welfare Survey, a biennial national household survey conducted by the National Statistical Office since 2017. The 2019 survey took place in March, through face-to-face interviews with 27,900 Thai adults aged 15 years or above who participated in the survey and compares 2019 findings with those from 2017.

**Results:**

One month prior to the survey, 6.3% of population reported use of antibiotics (reduced from 7.9% to 2017), of which 98.1% received antibiotics through healthcare professionals and almost half (43.2%) for flu symptoms. During the last 12 months, 21.5% of Thai adults received information on the appropriate use of antibiotics and AMR (increased from 17.8% to 2017); mostly through health professionals (82.7%). On knowledge, 24.3% of adults gave correct answers to more than three out of six statements (three true and three false statements) (increased from 23.7% to 2017). The overall mean score of awareness of appropriate antibiotic use and AMR is 3.3 out of total score of 5.

**Conclusions:**

Although progress was made on knowledge and awareness between 2017 and 2019, certain practices, such as use of antibiotics for flu symptoms and receiving information about antibiotic use and AMR, are inappropriate and inadequate. These findings require significant action, notably strengthening health professionals’ ability to prescribe and dispense antibiotics appropriately and effective communication with patients. The government should promote specific information on rational use of antibiotics and AMR to specific target groups.

## Background

Antimicrobial resistance (AMR) is a global problem. It is one of the important consequences of irrational use of antibiotics by the general public and healthcare providers [1, 2]. The irrational use of antibiotics includes use of antibiotics without a prescription, sharing leftover antibiotics, lack of adequate education and training in health professionals, promotion of pharmaceutical companies, and patient-doctor interactions. However, the common factor leading to irrational antibiotic use by the general public and healthcare providers is a low level of knowledge and awareness of antibiotic use and AMR [2].

Since 2002, the whole Thai population is covered by health insurance schemes. All medicines including antibiotics listed on the National Essential Drug List are covered in the benefit package [3]; this means patients do not pay upfront and providers are reimbursed as insurance schemes either prepay the providers using capitation or conduct retrospective reimbursement. Self-medication at private pharmacies is not covered by the health insurance benefit package and is not reimbursable. Almost all antibiotics can be accessed at licensed pharmacies and must be dispensed by licensed pharmacists, but some antibiotics classified by Thailand Food and Drug Administration as “specially controlled” drugs require a prescription from a physician/dentist/veterinarian to initiate the dispensing cascade [3]. Doctors in all private clinics also dispense medicines including antibiotics. At primary healthcare centers where there is no physician, in practice, professional nurses can dispense certain items of antibiotics.

To monitor public knowledge and awareness of antibiotic use and AMR, the World Health Organization (WHO), in collaboration with partners, developed a survey tool and conducted a survey in 12 countries of six regions in 2015 [4]. Flagging the importance of public knowledge and awareness in ensuring the appropriate use of antibiotics, in 2009 the European Commission launched a survey by applying the same methodology to monitor public knowledge and awareness called “Eurobarometer” [5]. Later, many countries such as Saudi Arabia, Sweden, Japan, Thailand, and Kosovo also launched their own survey [5]. Overall results revealed that the main factors associated with low levels of public knowledge and awareness on antibiotic use and AMR were education and wealth [6–10]. In Japan, 80% of the participants did not know that antibiotics do not kill viruses and that antibiotics are ineffective against cold and flu [7]. One of the five goals of the Thai National Strategic Plan on AMR (NSP-AMR)(2016-2021) is a 20% increase in public knowledge and awareness on antibiotic use and AMR by 2021 [11]. To implement a monitoring and evaluation platform for the goal, since 2017 an AMR module has been integrated into the Health Welfare Survey (HWS), which is a national biennial survey platform operated by the National Statistical Office (NSO). To ensure scientific validity and policy coherence, the module has also been regularly updated by the International Health Policy Program (IHPP) [12].

Despite regular updates to the platform and results since 2017, some challenges remain such as low levels of public knowledge on antibiotic use and AMR, low levels of exposure to information about symptoms and inappropriate use of AMR, and a lack of sufficient evidence to support effective interventions for relevant organizations [9]. As a result, this study aims to explore Thailand’s practices on antibiotic use, level of knowledge and awareness of antibiotic use and AMR and the factors associated with knowledge and awareness on antibiotic use and AMR for monitoring the national goal in 2019. This study compares findings with those in 2017.

## Methods

### Survey instrument

The AMR module was developed in the Thai language in consultation with a network on Health Policy and Systems Research on Antimicrobial Resistance (HPSR-AMR) in Thailand. The questionnaire was modified from the “Antibiotic resistance: multi-country public awareness survey” [4] and “Antimicrobial Resistance Eurobarometer Survey” [5] with additional questions specifically designed to suit the national context.

In the 2019 survey module, four sections with 17 questions collected respondents’ information on the use of antibiotics, their knowledge of antibiotics, their awareness of appropriate antibiotic use and AMR, and their reception of information on the use of antibiotics and AMR. The first section explored the use of antibiotics in the last month, the sources of antibiotics and the reasons for taking them. In section two, to assess the knowledge of antibiotics, statements were asked by using true or false statements and one question. In section three, five questions about awareness of appropriate antibiotic use and AMR used a five-point Likert-style response option, from strongly disagree to strongly agree. To ensure the accuracy of ‘yes’ and ‘no’ answers and a Likert-style response, a ‘do not know’ answer option was also provided. Section four asked respondents about whether they had received information on antibiotics and AMR during the last twelve months and the sources of such information (Table 1).

There was an additional section in the 2019 questionnaire, which concerned awareness of the importance of appropriate antibiotic use and AMR (Sect. 3 in Table 1). In addition, three questions were revised from the 2017 survey including AB3, AB4 and AB5_5 (Table 1). This module for the 2019 survey has been integrated into the national Health and Welfare Survey conducted by the National Statistical Office in 2019, which facilitates the comparison between 2017 and 2019.

**Table 1:** AMR module embedded in 2019 HWS

	Contents	Choices of answer
**I. USE OF ANTIBIOTICS, SOURCE OF ANTIBIOTICS AND REASON FOR TAKING ANTIBIOTICS**		
AB1	Have you taken any antibiotics orally such as tablets, powder or syrup in the last month?	Yes, No, Do not know
AB2 (IF ‘YES’ to AB1)	Where did you obtain the last course of antibiotics that you used?	**Choices of answer**: Health center, Community hospital, General or regional hospital, University hospital, Other public hospital, Private hospital, Private clinic, Pharmacy, Online, Grocery store, Left over antibiotics from the previous treatment (your own and others), Mobile medical Unit, Others (Specify)
fAB3 (IF ‘YES’ to AB1) ^a^	What were the symptoms for last taking the antibiotics that you used? (Multiple answers possible)	**Choices of answer**: Sore throat, Cough, Fever, Loose stool, Headache, Muscle aches, Pustule/purulent wound, Fresh wound/bleeding wound, Dysuria, Leukorrhea, Toothache, Others (Specify), No symptom, Do not know
AB4 (IF ‘YES’ to AB1) ^a^	What were the illnesses for last taking the antibiotics that you used? (Multiple answers possible)	**Choices of answer**: Pneumonia, Bronchitis, Pharyngitis/tonsillitis, Flu/cold, Watery Diarrhea, Bloody diarrhea/dysentery, Skin infection/wound infection, Cystitis/pyelonephritis, Vaginitis/pelvic inflammatory disease, Acute otitis media/sinusitis, Gingivitis/periodontitis, Others (Specify), No illness, Do not know
**II. KNOWLEDGE ABOUT ANTIBIOTIC USE AND AMR**		
AB5_1	Please tell me whether you think it is true or false. “Antibiotics kill viruses” (The correct answer is a false statement.)	True, False, Do not know
AB5_2	Please tell me whether you think it is true or false. “Antibiotics are effective against colds and flu” (The correct answer is a false statement.)	True, False, Do not know
AB5_3	Please tell me whether you think it is true or false. “Unnecessary use of antibiotics makes them become ineffective or induces bacterial resistance” (The correct answer is a true statement.)	True, False, Do not know
AB5_4	Please tell me whether you think it is true or false. “Taking antibiotics often has side-effects such as diarrhea” (The correct answer is a true statement.)	True, False, Do not know
AB5_5 ^a^	Please tell me whether you think it is true or false. “Antibiotics are equal to anti-inflammatory drugs” (The correct answer is a false statement.)	True, False, Do not know
AB6	When do you think you should stop taking antibiotics once you have begun a course of treatment?	**Choices of answer**: When your illness is better, When you get full course of antibiotics (from doctor’s or health professionals recommendation), Others (Specify), Do not know
**III. AWARENESS OF THE IMPORTANCE OF APPROPRIATE ANTIBIOTIC USE AND AMR** ^**a**^		
AB7_1	How much do you agree with following statements: People should use antibiotics only when they are prescribed by a doctor, nurse or pharmacist	Strongly disagree, Slightly disagree, Neither agree nor disagree, Slightly agree, Strongly agree, Do not know
AB7_2	How much do you agree with following statements: People should not keep antibiotics and use them later for other illnesses	Strongly disagree, Slightly disagree, Neither agree nor disagree, Slightly agree, Strongly agree, Do not know
AB7_3	How much do you agree with following statements: Antibiotic resistance is one of the problems that should be considered	Strongly disagree, Slightly disagree, Neither agree nor disagree, Slightly agree, Strongly agree, Do not know
AB7_4	How much do you agree with following statements: I am worried about the impact that antibiotic resistance will have on my health, and that of my family	Strongly disagree, Slightly disagree, Neither agree nor disagree, Slightly agree, Strongly agree, Do not know
AB7_5	How much do you agree with following statements: I am not at risk of getting an antibiotic resistant infection, as long as I take my antibiotics correctly	Strongly disagree, Slightly disagree, Neither agree nor disagree, Slightly agree, Strongly agree, Do not know
**IV. PUBLIC INFORMATION ABOUT THE APPROPRIATE USE OF ANTIBIOTICS AND AMR**		
AB8	In the last 12 months, do you remember getting any information about not taking antibiotics unnecessarily, for example for a cold or the flu, or information on antimicrobial resistance?	Yes, No, Do not know
AB9 (IF ‘YES’ to AB8)	Whom did you get this information about not taking antibiotics unnecessarily?	**Choices of answer**: Leaflet/poster, Newspaper, Radio, TV, Internet/social media, Family members/Friends, Doctor, Nurse, Pharmacist, Other health professionals, Others (Specify), Do not know

^a^ This section was revised or not included in the 2017 survey

The questionnaire was peer reviewed by four external experts from different fields (pharmacologist, public health specialists and health promotion specialists) for content validity, the logic and clarity of the content. The questionnaire was then submitted in a pilot test to thirty individuals, which were randomly selected from lay people in Saraburi Province to check reliability and clarity of the questions. After piloting, the questionnaire was revised based on experts’ opinions and pilot tested.

### Sampling and Data Collection

The AMR module was embedded in a national representative cross-sectional household interview survey called the Health and Welfare Survey, which is carried out biennially by the National Statistical Office (NSO). The survey was conducted in March 2019 across 77 provinces in Thailand.

The same sampling method in 2017 HWS was applied in the 2019 HWS. The HWS applied a stratified two-stage sampling. Greater Bangkok and the remaining provinces constituted strata, with 77 strata altogether. Each stratum was divided into municipal (urban) and non-municipal (rural) areas. In the first stage, sampling enumeration areas (EAs) from urban and rural areas were selected using probability proportional to size based on total household numbers. In the second stage, the sampling units were private households. In each sampled EA, a systematic random sample of private households was selected [9]. In total 27,960 sample households were calculated for the HWS 2019. Of these, only 23,549 households participated with an overall response rate of 84.2%. The remaining were empty houses or could not be identified due to errors in addresses. Of these 23,549 households, a total of 63,594 members of all ages participated in the HWS.

Of the total 63,594 household members, only 27,900 adult members met the eligibility criteria to answer the AMR module. They were 15 years old or above and presented themselves on the survey’s date and time. We assumed that at least one respondent per household in the total 27,960 households meet the eligible criteria, then the estimate response rate of the AMR module would be 99.8% (27,900/27,960).

Before starting the survey, all interviewers from the NSO across 77 provinces were trained via a video conference. The AMR interview guidelines were produced by researchers and provided to the interviewers to ensure high quality, reliable and valid information was collected from the survey, such as the definition of antibiotics and antimicrobial resistance. For example, we explained that antibiotics means “drug used for stopping or killing microbes that cause infections in humans and animals” and AMR means “when a patient is affected by AMR pathogens, various antibiotics are not able to kill them and effective treatment results in mortality”. The interviewers would also pass on this information to the respondents. The interview was conducted in Thai and took about 70 min. Data were collected by interviewers using a software program developed by the NSO. All data were anonymous; a tracking number was used for each respondent to ensure confidentiality.

The study was approved by the Institute for the Development of Human Research Protections for research ethics clearance (Ref.no.IHRP2019057).

### Data analysis

Data were analyzed using STATA/IC (version 14.2). Descriptive measures were presented according to population weight number and percentages. Differences in distribution between groups were compared with an estimate of 95% confidence intervals (CIs). For all tests, p-values of 0.05 or less were considered to be significant. The multivariate logistic regression analysis with Likelihood Ratio test was used to determine a relationship between demographic variables (sex, age, education level, area of residence and wealth quintile) and receiving information on use of antibiotics and AMR, levels of knowledge and awareness of antibiotic use and AMR that were of significant bivariate association (Chi-square test).

The cutoff points were used in the analysis for the outcome variable of levels of knowledge and awareness of antibiotic use and AMR. The knowledge score was calculated from one point of each statement to a total of six points. Average awareness score was analyzed from five awareness statements ranged from one to five of the Likert scale including the reverse scale of AB7_2 and AB7_5. The cutoff point for levels of knowledge and awareness was set at more than 60%. Equal to or lower than three was defined as low knowledge or less aware, and higher than three as high knowledge or more aware [13, 14].

Three questions in 2019 were revised from the 2017 version. The AB3 and AB4 were designed to identify reasons for using antibiotics for a range of symptoms (AB3) and diseases (AB4). The previous question in the 2017 version was: ‘What was the reason for your last taking the antibiotics?’. Choices of answers were pneumonia, bronchitis, rhinitis and rhinopharyngitis, flu/influenza, sore throat, cough, fever, headache, diarrhea, urinary tract infection, skin or wound infection and others. The researchers felt the reasons for antibiotics use in 2017 version could not accommodate the use of antibiotics because 17.1% of respondents answered ‘others’ as their indication of antibiotic use. In addition, the validity of self-reporting antibiotic indications was proved reliable in a previous study due to the similarity with prescription data such as respiratory tract infections, ear infections and urinary tract infections [15]. Moreover, to prevent misunderstandings in asking negative questions, in 2019 a version of the AB5_5 was reformulated from ‘Antibiotics are not equal to anti-inflammatory drugs (a true statement)’ used in 2017 version, to ‘Antibiotics are equal to anti-inflammatory drugs (a false statement)’. The negative statement in 2017 version became a positive statement in 2019. Answers in both rounds were evaluated as correct and incorrect answers.

## Results

### Antibiotic use in the past month, sources, and reasons for taking antibiotics

**Fig. 1:**
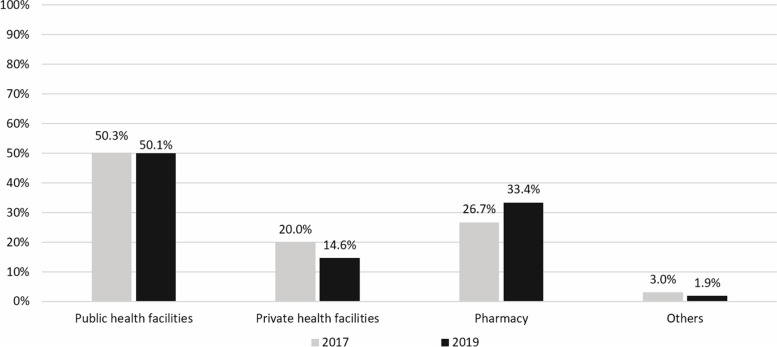
Percentage of respondents who used antibiotics in the last month classified by source of antibiotics: comparative findings between 2017 and 2019

In 2019, of the total respondents, 6.3% reported use of antibiotics in the month prior to the survey; while 10.1% of respondents could not confirm whether the medicines they used were antibiotics or otherwise. Among those who reported antibiotics use, the vast majority (98.1%) obtained the last course of antibiotics from a healthcare professional including 50.1% from public healthcare facilities, 33.4% from private pharmacies and 14.6% from private healthcare facilities. A minority of respondents, 1.9% said they obtained antibiotics from grocery stores, used leftover medicines from a previous course or purchased antibiotics online (Fig. 1).

**Fig. 2:**
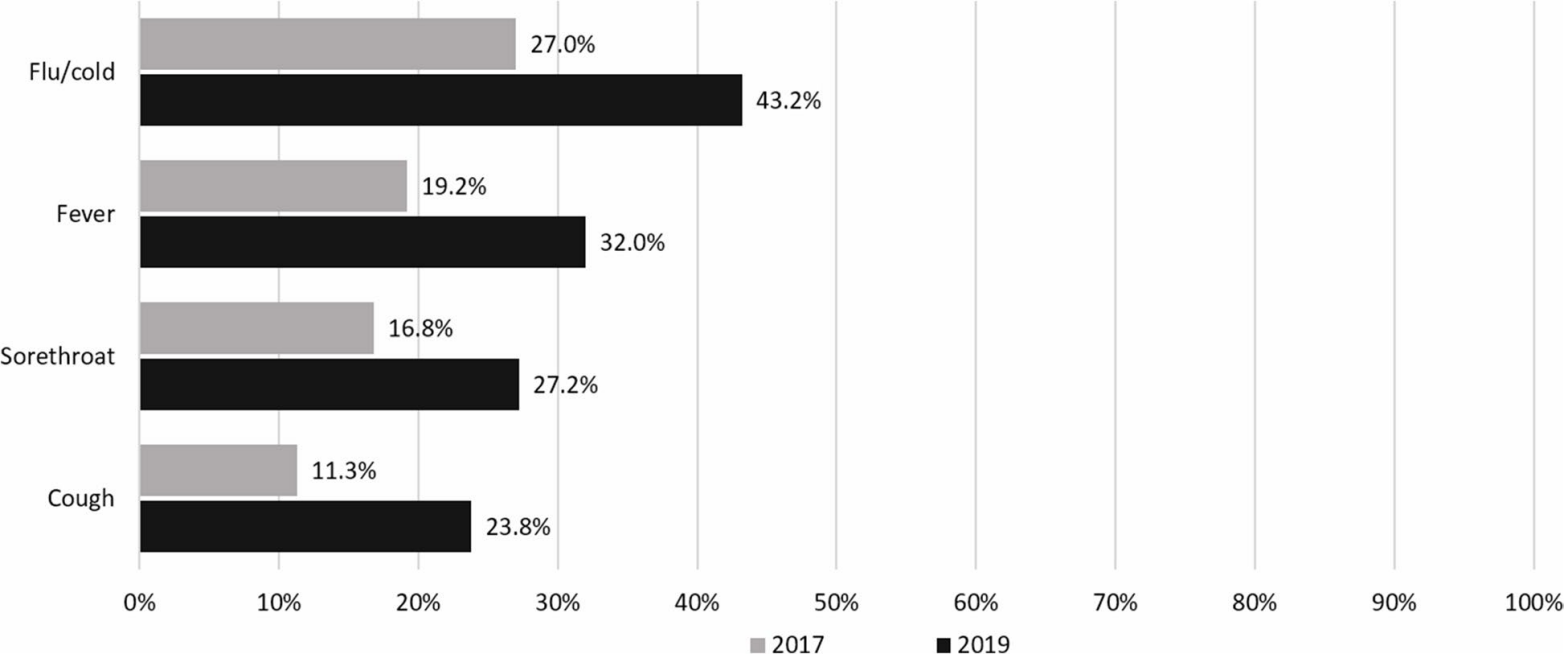
Percentage of respondents who used antibiotics in the last month classified by reasons for taking antibiotics: comparative findings between 2017 and 2019. Note: Total percentages were more than 100% due to multiple answers

In 2019, of the self-reported clinical indications for antibiotic use, the most common reason of illness was flu (43.2%) and the most common symptoms for taking antibiotics were fever (32.0%), sore throat (27.2%) and cough (23.8%) (Fig. 2). When compared with the 2017 HWS, there has been an increase in the proportion of people taking antibiotics for flu (+16.2% point change) followed by fever (+12.8% point change), cough (+12.5% point change) and sore throat (+10.4% point change). It is interesting that 14.7% of respondents said they did not have any illnesses when they took antibiotics. Other symptoms or illnesses such as muscle aches and pharyngitis, etc. ranged from 0.2 to 14.4% in 2019.

### 2. Public information on appropriate use of antibiotics and AMR

**Fig. 3:**
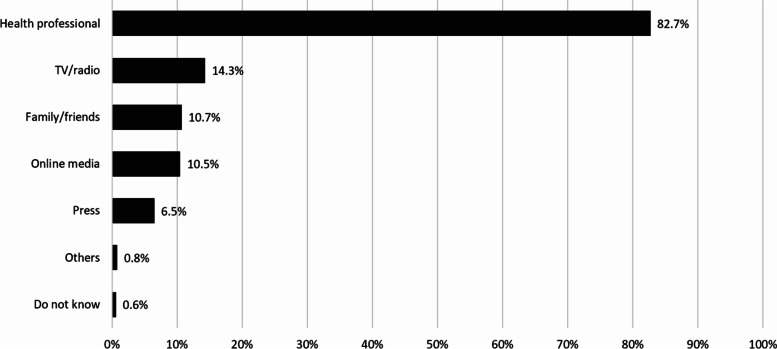
Percentage of respondents who received information on appropriate use of antibiotics and AMR in 2019, classified by sources of information. Note: Total percentages were more than 100% due to multiple answers

Of respondents, 21.5% recalled receiving information about appropriate use of antibiotics and AMR in the last 12 months; for which the most common sources of information were health professionals (82.7%). Other sources played a minor role including television and radio (14.3%), family and friends (14.3%) and online media (10.7%). Other sources such as newspapers, posters, and leaflets were insignificance (Fig. 3).

### Knowledge about appropriate antibiotic use and AMR

**Fig. 4:**
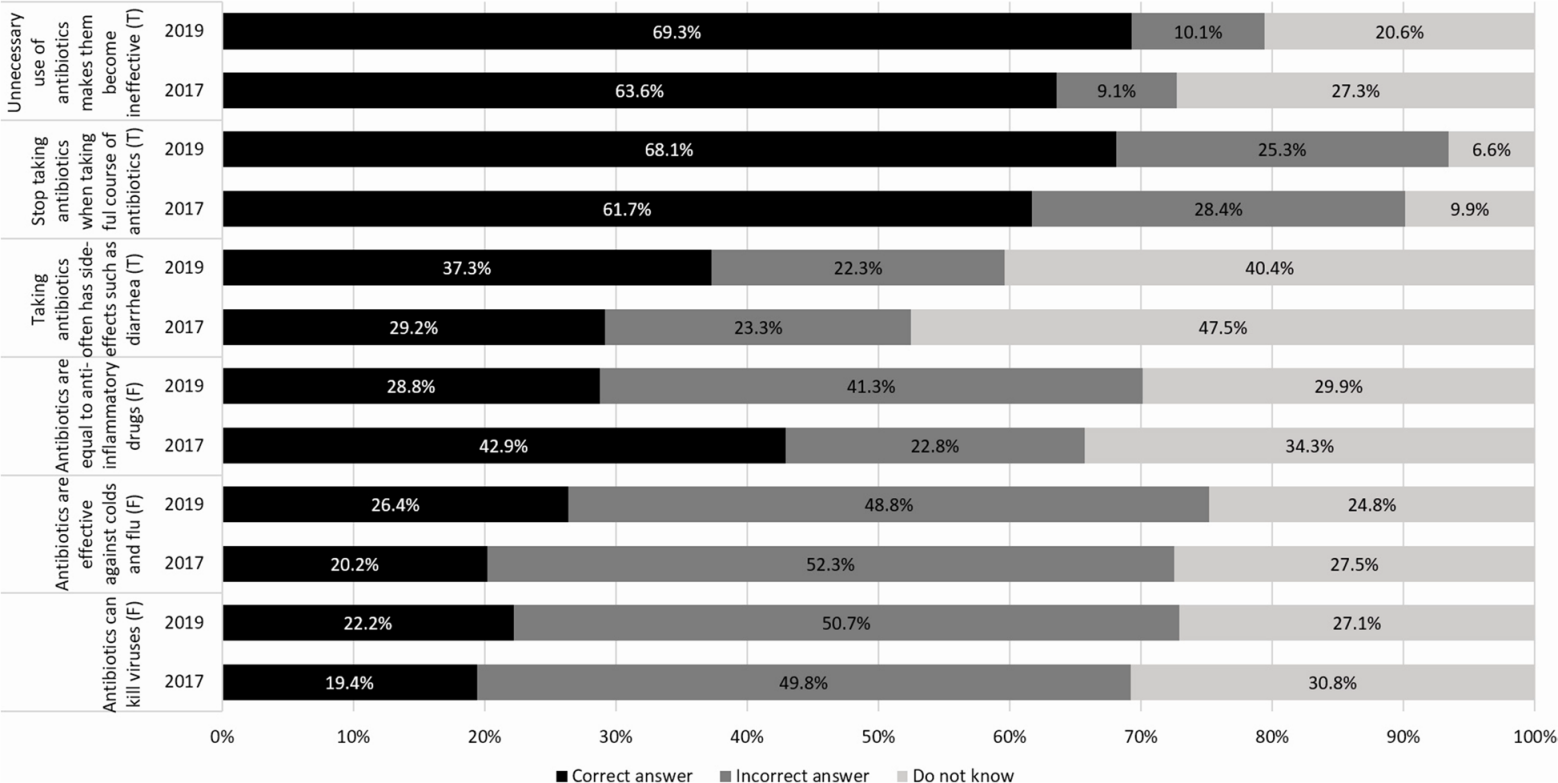
Percentage of respondents who gave correct answers in each statement of knowledge on antibiotic use: comparative findings between 2017 and 2019. Note: T refers to true statement and F false statement

A majority (>65%) answered correctly to the two questions regarding antibiotic use: “unnecessary or inappropriate use of antibiotics makes them become ineffective or induces bacterial resistance” and “antibiotic treatment should only be stopped when the whole course of antibiotics has been taken as directed”. Fewer respondents incorrectly answered that “antibiotics can kill viruses” (50.7%); “antibiotics are effective for treatment of colds and flu” (48.8%); and “antibiotics are equivalent to anti-inflammatory drugs” (41.3%). Of respondents, 40.4% did not know the side effects of using antibiotics such as diarrhea. The proportion of respondents who gave correct answers to knowledge statements in 2019 was higher in the 2017 survey except the statement about “antibiotics and inflammatory drugs” which changed from 42.9 to 28.8% of correct answers (Fig. 4).

**Fig. 5:**
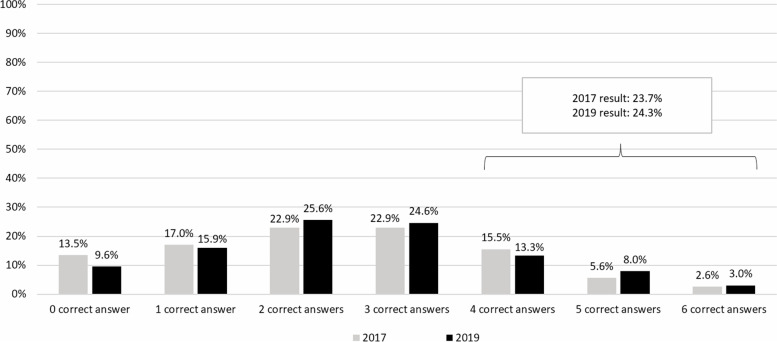
Percentages of respondents who gave correct answers between 2017 and 2019

In 2019, only 3.0% of respondents correctly answered all six questions and about a quarter of respondents (24.3%) gave more than three correct answers. However, 9.6% of respondents gave wrong answers to all six statements (Fig. 5). The mean score of correct answers was 2.5 out of the total 6 scores (SD=1.5). Comparing data between 2017 and 2019, there was an increasing trend of people who gave correct answers equal to and more than two score points. Specifically, levels of knowledge on AMR and antibiotic use have slightly increased from 23.7% to 2017 to 24.3% of adults who gave correct answers to more than three out of six true/false statements in 2019.

#### Awareness of appropriate antibiotic use and antimicrobial resistance

**Fig. 6:**
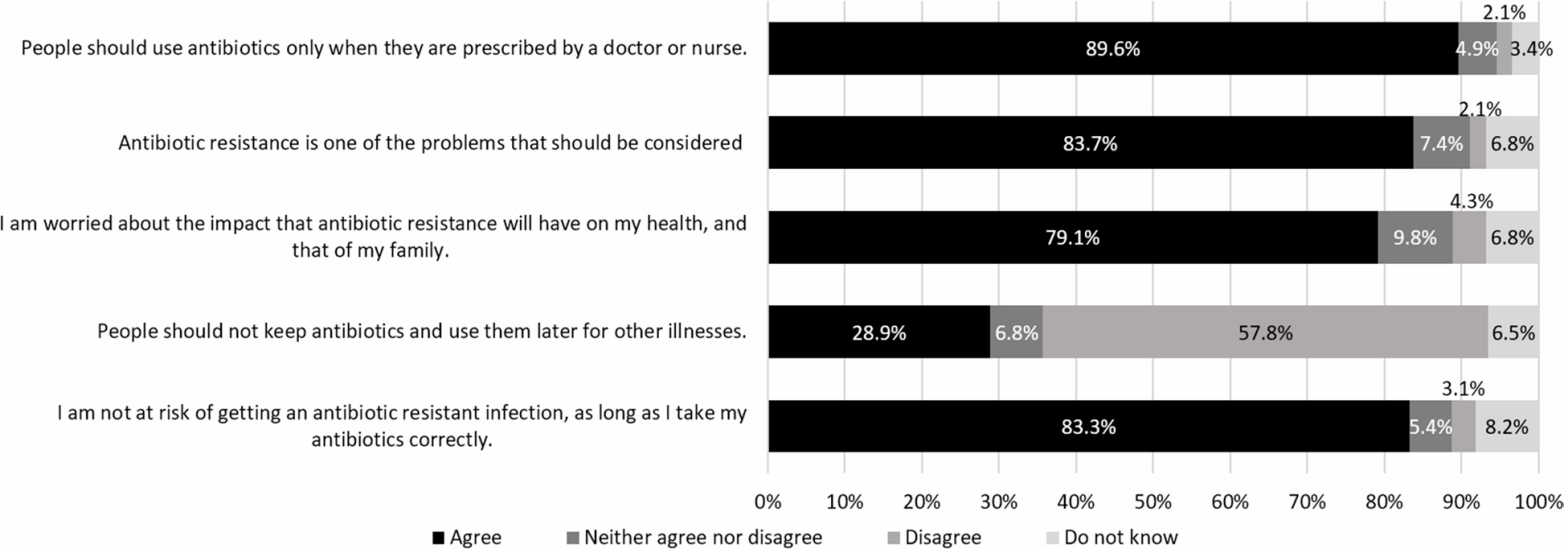
Level of agreement by respondents on five statements on awareness of appropriate antibiotic use and AMR in 2019

The majority of respondents agreed that people should use antibiotics only when they are prescribed by a doctor or nurse (89.6%); 83.7% agreed that antibiotic resistance is one of the problems that should be considered; 79.1% agreed with the statement ‘I am worried about the impact that antibiotic resistance will have on my health, and that of my family’ and 57.8% disagreed with the statement that people should keep antibiotics and use them later for other illnesses (Fig. 6). However, 83.3% believed that they are not at risk if they use antibiotics as prescribed, although this is not in fact the case. The overall mean score of the adult population who are aware of the importance of antibiotic use and AMR is 3.3 out of maximum score of 5 (SD=0.8).

### Factors associated with access to information, knowledge and awareness: a multivariate analysis

The respondents’ characteristics associated with access to information, knowledge and awareness are shown in Table 2.

**Table 2:** Multivariate analysis of characteristics of respondents associated with receiving information about antibiotic use and AMR, knowledge, and awareness of antibiotic use and AMR

Characteristic	Public information on appropriate use of antibiotics and AMR					Knowledge about appropriate antibiotic use and AMR					Awareness of appropriate antibiotic use and AMR				
	Number of respondents who did not receive information (%)	Number of respondents who received information (%)	Adjusted OR	95% CI	P-value	Number of respondents with ≤ 3 correct answers (%)	Number of respondents with > 3 correct answers (%)	Adjusted OR	95% CI	P-value	Number of respondents with awareness score ≤ 3 (%)	Number of respondents with awareness score > 3 (%)	Adjusted OR	95% CI	P-value
**Gender**					**<0.001**^**a**^ **(LR test)**					**0.097**					**0.011**^**a**^ **(LR test)**
Male	8,893 (41.4)	2,302 (36.0)	Reference			8,654 (39.9)	2,541 (41.0)	Bivariate analysis is not statistically significant			2,348 (41.3)	8,847 (39.8)	Reference		
Female	12,608 (58.6)	4,097 (64.0)	1.26	1.19-1.34	<0.001^**a**^	13,054 (60.1)	3,651 (59.0)				3,333 (58.7)	13,372 (60.2)	1.08	1.02-1.15	0.011^**a**^
**Age group, years**					**<0.001**^**a**^ **(LR test)**					**<0.001**^a^ **(LR test)**					**<0.001**^a^ **(LR test)**
15-24	1,105 (5.1)	273 (4.3)	Reference			1,042 (4.8)	336 (5.4)	Reference			278 (4.9)	1,100 (5.0)	Reference		
25-59	13,153 (61.2)	3,957 (61.8)	1.19	1.03-1.37	0.016^**a**^	12,912 (59.5)	4,198 (67.8)	1.07	0.94-1.22	0.322	3,131 (55.1)	13,979 (62.9)	1.22	1.05-1.40	0.007^**a**^
≥ 60	7,243 (33.7)	2,169 (33.9)	1.30	1.12-1.51	0.001^**a**^	7,754 (35.7)	1,658 (26.8)	0.87	0.76-1.01	0.064	2,272 (40.0)	7,140 (32.1)	1.02	0.88-1.19	0.749
**Area of residence**					**0.177**					**0.546 (LR test)**					**0.002**^a^ **(LR test)**
Urban	11,857 (55.2)	3,590 (56.1)	Bivariate analysis is not statistically significant			11,870 (54.7)	3,577 (57.8)	Reference			2,917 (51.3)	12,530 (56.4)	Reference		
Rural	9,644 (44.8)	2,809 (43.9)				9,838 (45.3)	2,615 (42.2)	1.02	0.96-1.08	0.545	2,764 (48.7)	9,689 (43.6)	0.91	0.86-0.96	0.002^a^
**Education level**					**<0.001**^**a**^ **(LR test)**					**<0.001**^**a**^ **(LR test)**					**<0.001**^**a**^ **(LR test)**
Uneducated	1,122 (5.2)	190 (3.0)	Reference			1,154 (5.3)	158 (2.6)	Reference			449 (7.9)	863 (3.9)	Reference		
Primary school	12,282 (57.1)	3,474 (54.3)	1.58	1.35-1.85	<0.001^a^	12,982 (59.8)	2,774 (44.8)	1.40	1.18-1.66	<0.001^a^	3,652 (64.3)	12,104 (54.5)	1.51	1.34-1.71	<0.001^a^
Secondary school	5,994 (27.9)	1,835 (28.6)	1.73	1.46-2.04	<0.001^a^	5,704 (26.3)	2,125 (34.3)	2.16	1.81-2.59	<0.001^a^	1,245 (21.9)	6,584 (29.6)	2.18	1.90-2.50	<0.001^a^
University and above	2,063 (9.6)	895 (14.0)	2.16	1.80-2.59	<0.001^a^	1,829 (8.4)	1,129 (18.2)	3.27	2.70-3.97	<0.001^a^	318 (5.6)	2,640 (11.9)	3.03	2.54-3.61	<0.001^a^
Others and unknown	40 (0.2)	5 (0.1)	0.89	0.35-2.29	0.809	39 (0.2)	6 (0.1)	1.11	0.46-2.67	0.816	318 (0.3)	2,640 (0.1)	0.88	0.48-1.63	0.688
**Wealth quintile**					**<0.001**^**a**^ **(LR test)**					**<0.001**^**a**^ **(LR test)**					**<0.001**^**a**^ **(LR test)**
Q1 (poorest)	5,298 (24.6)	1,177 (18.4)	Reference			5,448 (25.1)	1,027 (16.6)	Reference			1,825 (32.1)	4,650 (20.9)	Reference		
Q2	4,725 (22.0)	1,276 (19.9)	1.16	1.06-1.26	0.001^a^	4,789 (22.1)	1,212 (19.6)	1.25	1.14-1.37	<0.001^a^	1,226 (21.6)	4,775 (21.5)	1.41	1.29-1.53	<0.001^a^
Q3	4,293 (20.0)	1,331 (20.8)	1.31	1.20-1.44	<0.001^a^	4,424 (20.4)	1,200 (19.4)	1.28	1.17-1.41	<0.001^a^	1,079 (19.0)	4,545 (20.5)	1.48	1.36-1.62	<0.001^a^
Q4	4,107 (19.1)	1,400 (21.9)	1.39	1.27-1.52	<0.001^a^	4,424 (18.8)	1,200 (22.9)	1.48	1.35-1.63	<0.001^a^	949 (16.7)	4,558 (20.5)	1.55	1.41-1.69	<0.001^a^
Q5 (wealthiest)	3,078 (14.3)	1,215 (19.0)	1.49	1.35-1.65	<0.001^a^	2,958 (13.6)	1,335 (21.5)	1.57	1.42-1.75	<0.001^a^	602 (10.6)	3,691 (16.6)	1.66	1.49-1.86	<0.001^a^
Total	21,501 (100.0)	6,399 (100.0)				21,708 (100)	6,192 (100.0)				5,681 (100.0)	22,219 (100.0)			

Likelihood Ratio test (LR test) evaluates the difference nested models in which one model restricts a parameter to zero by removing the predictor variables from the model

^a^Refer to statistically significant at *P*-value <0.05

### Public information on appropriate use of antibiotics and AMR

The multivariate analysis using logistic regression showed that respondents who were female, older age, having higher education and in a richer wealth quintile were more likely to receive information on appropriate antibiotic use and AMR. Females had a 1.3 times higher chance than males to receive public information about antibiotics and AMR (OR=1.26; 95%CI =1.19-1.34; p-value<0.001). The older the respondents, the higher their opportunity to receive information related to antibiotics and AMR (OR=1.30; 95%CI =1.12-1.51; p-value<0.001). Moreover, the respondents who belonged to the highest education level and richest wealth quintile were 2.2-fold and 1.5-fold more likely to receive public information about antibiotics and AMR than those in the uneducated level (OR=2.16; 95%CI =1.80-2.59; p-value<0.001) and the poorest quintile (OR=1.49; 95%CI =1.35-1.65; p-value<0.001).

### Knowledge about appropriate antibiotic use and AMR

The respondents with higher education and in richer wealth quintiles were more likely to have higher knowledge on appropriate antibiotic use and AMR. Respondents who had a bachelor degree or higher were 3.3-fold more likely to have better knowledge than those who were uneducated (OR=3.27; 95%CI =2.70-3.97; p-value<0.001) and those who were in the highest wealth quintile were 1.6-fold more likely to have better knowledge than those in the lowest wealth quintile (OR=1.57; 95%CI =1.42-1.75; p-value<0.001).

### Awareness of appropriate antibiotic use and AMR

The respondents who were female, adults, living in urban areas, with higher education and in a richer wealth quintile were more likely to be aware of appropriate antibiotic use and AMR. Females were 1.1 times more likely to be aware of appropriate antibiotic use and AMR than males (OR=1.08; 95%CI =1.02-1.15; p-value=0.011) and adults were 1.2 times more likely to be aware than adolescents (OR=1.22; 95%CI =1.05-1.40; p-value=0.007). 9% of respondents who lived in rural areas were less aware of appropriate antibiotic use and AMR compared to those who lived in urban areas (OR=0.91; 95%CI =0.86-0.96; p-value=0.002). Moreover, respondents who had a bachelor degree or higher were three times more likely to be aware of appropriate antibiotic use and AMR than those who were uneducated (OR=3.03; 95%CI =2.54-3.61; p-value<0.001) while those who belonged to the richest wealth quintile had a 1.7 times higher chance of being aware about antibiotic use and AMR than the poorest quintile (OR=1.66; 95%CI =1.49-1.86; p-value<0.001).

## Discussion

### Antibiotic use in the past month, sources, and reasons for taking antibiotics

The number of Thai adults reporting they had taken antibiotics during the last month decreased from 7.9% to 2017 to 6.3% in 2019 [9]. In comparison with other studies, the use of antibiotics during the last month among the general population in Thailand was lower than in international peers such as Malaysia (16.5%), which conducted its survey in March; the same month of this study [16].

Major challenges include that 10.1% of respondents were uncertain whether the medicines they took in the last month were antibiotics or not. Different Thai words can refer to “antibiotic” such as y*aa-kha-chue* (“drug that kills germs”), y*aa-khae-akseab* (“anti-inflammatory drug”) and *yaa-pati-cheewana* (“drug that fights microbes”) [17]. A study among 12,868 Thai elderly people showed that only 6% of them correctly named antibiotics while others did not know or used different names such as y*aa-khae-akseab* (“anti-inflammatory drug”) [18]. The proper Thai word for “antibiotic” should be applied in effective communication.

This study shows that people in Thailand mainly received antibiotics from health professionals at 98.1% which is higher than the findings from the Eurobarometer survey (93.0%), South Africa (93.0%), Mexico (92.0%) Vietnam (75.0%), Indonesia (83.0%) and the Russian Federation (56.0%) [5]. This means, in Thailand, health personnel are key change agents who can promote appropriate antibiotic use in the community.

Despite the high professional source of antibiotics, 43.2% of respondents said they used them for flu. There is an urgent need to assess, monitor and strengthen health professional’s antibiotic competency through pre- and in-service training. Antibiotic stewardship should not only focus on physicians but also other health professionals such as pharmacists and nurses who dispense antibiotics.

### Public information on appropriate use of antibiotics and AMR

This survey shows that the coverage of public information on antibiotics and AMR is inadequate; less than a quarter of respondents (21.5%) had received information in the past 12 months. Though the rate is slightly higher than in Malta (18.0%) and Croatia (18.0%), it is much lower than in Finland (59.0%), Sweden (47.0%), France (45.0%), Belgium, Germany and the UK (43.0% each) [5]. Health professionals (doctors, nurses and pharmacists) are the most common source of information in Thailand (82.7%), similar to European countries [19, 20]. The high level of population trust in health professionals [21] is the social capital for appropriate use of antibiotics and AMR awareness campaigns through healthcare workers [22]. The NSP-AMR need to re-orient its interventions by strengthening health professionals’ antibiotics stewardship and competency as these people can serve as change agents for AMR awareness in the population in addition to general communication campaigns in the population [3].

### Knowledge about appropriate antibiotic use and AMR

The 2019 survey shows that Thai respondents have limited knowledge about antibiotics inability to kill viruses. Thailand has a 22.2% rate, compared with a 43.0% average across 29 EU member states, although Thailand is on par with Greece at 23%, which is the lowest level among EU, and Japan at 22% [5, 7]. Knowledge that antibiotics are not effective against colds and flu is 26.4% in Thailand compared with a 66.0% average for the EU, and 37% in Portugal, which is the lowest level in EU, although Thailand is again on a par with Japan’s 24.6% [5, 7].

The improved proportion of respondents who gave correct answers to knowledge statements is a result of campaigning during the 2018 antibiotic awareness week which promoted ‘no antibiotic use for flu’ [23]. The focus of further effective communication should be that antibiotics do not kill viruses.

### Awareness of appropriate antibiotic use and antimicrobial resistance

This study shows high levels of awareness about appropriate use of antibiotics and AMR (around 80%) except for the use of left-over antibiotics. More than half of respondents, 57.8%, agreed it was not good to use the left-over antibiotics, which is a lower figure than the 70% shown in a multi-country study [4]. Moreover, there was misunderstanding to the statement “I am not at risk of getting an antibiotic-resistant infection, as long as I take my antibiotics correctly”. Though this is a false statement, the majority of respondents in Thailand (83.3%) and in a multi-country survey (63.0%) believed it to be a true statement.

### Factors influencing access to information, knowledge and awareness: a multivariate analysis

Similar to the 2017 survey, education and wealth status were significantly associated with receiving public information, and a level of knowledge of appropriate antibiotic use and AMR [9]. A study in Japan found a positive association between high education levels and knowledge of AMR [24]. Similar findings from Norway indicated that lower educated and poorer people are targets for AMR communication [25]. Moreover, it is undeniable that education determines health literacy including use of antibiotics and AMR. A study in London showed 32% of people in affluent areas have more exposure to antibiotic campaigns than in deprived areas, at 17% [2]. Promoting appropriate antibiotic use can be successful when local context, target population and barriers are taken into account in the design [26].

This study shows that females were more likely to receive information about appropriate antibiotic use and AMR and have higher awareness than males. In many countries including Thailand, women are mainly decision-makers in health care and caregivers to at least two family members [27, 28]. Older individuals have more opportunities to be exposed to professional sources of AMR when seeking care at health facilities compared to adolescents. Hence, target interventions and empowering women and adults as key change agents in AMR is recommended [29].

### Monitoring of Thailand National Strategic Plan on AMR (2017-2021)

To measure progress towards NSP-AMR, the research team proposes three indicators, which are well accepted by the national steering committee on AMR. This includes: (a) percentage of Thai adults who provided correct answers to four out of the six true/false statements on appropriate antibiotic use and AMR; (b) mean score of Thai adults who are aware of the importance of appropriate antibiotic use and awareness of AMR (maximum 5 score); and (c) percentage of Thai adults who received information about AMR and appropriate antibiotic use in the last 12 months.

On indicator A, the percentage of adults who provide correct answers to more than 60% of the true/false statements minimally increased from 23.7% to 2017 to 24.3% in 2019 [9]; such minimal changes, 0.7% points, does not reach anywhere near the 20% increase as planned in the NSP-AMR. On indicator B, the mean score of the adult population who were aware of the importance of appropriate antibiotic use and AMR was 3.3 out of maximum 5 score; we cannot measure progress on indicator B as there was no 2017 baseline data. Only indicator C, the percentage of the adult population who received information about antibiotics and AMR during the last 12 months, had reached the NSP-AMR target; it increased from 17.8% to 2017 to 21.5% in 2019, a 20.8% increase [9].

### Key challenges and solutions

This study showed that health professionals are key sources of antibiotics and information, however it appears that antibiotics were frequently prescribed and dispensed for flu and common colds. Moreover, the respondents still have insufficient knowledge and awareness even after AMR campaigns that have been promoted for years.

Promoting rational use of antibiotics requires multiple interventions in a comprehensive manner, not only through citizen campaigning but through improving health workers’ prescribing and dispensing competency. Regulatory measures such as key performance indicators of the Ministry of Public Health (MOPH) are introduced to reduce antibiotics for normal birth delivery to below 10%, for upper respiratory tract infection and acute diarrhea to below 20% and traumatic wounds to below 40% [30]. These indicators are applied to MOPH health facilities but there is a room to expand in all public and private health facilities.

Moreover, health professionals may face pressures from patients demanding antibiotics [31]. Providing reminder messages before prescription, comparing antibiotics prescription rates with professional peers [32], and strengthening communication skills to manage patients’ demand for antibiotics from patients could all be tried as approaches.

### Strengths and limitation of study

The strength of this study is its large sample size and nationally representative data. Data were collected using the well-established survey methodology by the NSO. This is the second survey using the same methodology, which allows the monitoring of progress of the NSP-AMR. A major limitation of this study is the respondents’ capacity to distinguish antibiotic from other medicines and some evidence shows the low levels of understanding about antibiotics and AMR in respondents. Although the definition of antibiotics and AMR were fully provided in the training material and field manual where all interviewers were fully trained prior to the survey, challenges remained from both interviewers and respondents. Despite being trained, NSO interviewers are non-health personnel; a thorough understanding of antibiotics and capacity to describe to respondents can be challenging. However, a complete list of antibiotics that were common “lay-people” terms used by local communities is a helpful guide during the interview in future survey.

Health and Welfare Survey was conducted biennially by NSO in April; which is summer month in Thailand. The differences in survey period and seasonal variation of epidemiology of illness across studies hamper comparison. However, there is no empirical evidence on the seasonal variation of antibiotic use in the Thai population and it can be explored in future research.

A recall bias of receiving information on antibiotics and AMR is another limitation. Though no single recall period is best for all measures or all phenomena, the recall period must correspond to the characteristics of the phenomenon of interest and the purpose of the assessment [33]; Eurobarometer 478 applies a 12-months recall period in its survey for use of antibiotics and receiving information related to AMR [5]; while the WHO survey in 12 countries applies one- and six-months, one-year and more than one-year recall period for the use of antibiotics [4]. The researchers recognize and minimize recall bias by applying one-month for the use of antibiotics, which is similar to one-month prevalence of self-reported illness and healthcare utilization, applied by the national Health and Welfare Survey [34]. We applied a 12-months recall period for receiving information related to appropriate antibiotic use and AMR similar to Euro-barometer and country communication campaigns [3, 5].

### Conclusions and recommendations

This study compares findings from the 2017 with 2019 national survey. Knowledge about antibiotic use and AMR awareness was still low among respondents. Most people who received antibiotics in the previous month had inappropriate indication such as flu despite most antibiotics was provided by health professionals. The study shows fewer people had received antibiotics and AMR messages in 2019 than in 2017. Despite effort made by stakeholders as part of NSP-AMR to increase the public knowledge and awareness on antibiotic use and AMR in the population, the outcomes are not as expected especially the coverage of information, knowledge about appropriate antibiotic use and AMR. The continued inappropriate indications of antibiotic use prescribed by health professionals reflects either low level of antibiotic competency or lack of continued professional education and effective regulatory measures. Based on evidence from this study, we propose the following: (a) assess, monitor and strengthen health professionals’ antibiotics stewardship capacity where antibiotics are appropriately prescribed and dispensed; and introduce other effective regulatory measures (b) strengthen health professional communication skills as they are key change agents for increasing AMR awareness; and (c) use interventions to target specific groups with messages and communication to fill their knowledge gaps and concerns.

## Data Availability

The data that support the findings of this study are available from the National Statistical Office, but restrictions apply to the availability of these data, which were used under license for the current study, and so are not publicly available. Data are however available from the authors upon reasonable request and with permission of the National Statistical Office.
